# Identification of diagnostic blood indicators associated with adenomyosis: a retrospective cohort study

**DOI:** 10.3389/fendo.2026.1754036

**Published:** 2026-06-03

**Authors:** Yuehong Tong, Jiarong Luo, Yiye Chi, Xinyuan Cui, Jianlin Zhang, Hui Wang, Fang Yao

**Affiliations:** 1 Department of Gynecology, Jinhua Maternal and Child Healthcare Hospital, Jinhua, Zhejiang, China; 2 Institute of Maternal and Child Medicine Research, Shenzhen Maternity and Child Healthcare Hospital, Southern Medical University, Shenzhen, Guangdong, China; 3 Shenzhen Key Laboratory of Maternal and Child Health and Diseases, Shenzhen, Guangdong, China; 4 Bachelor Program of Bioinformatics, School of Medicine, The Chinese University of Hong Kong, Shenzhen, Guangdong, China; 5 Department of Gynecology, Shenzhen Maternity and Child Healthcare Hospital, Southern Medical University, Shenzhen, Guangdong, China

**Keywords:** adenomyosis, blood indicators, diagnosis, logistic regression, retrospective cohort study

## Abstract

**Background:**

Adenomyosis (AM) is described as a benign invasion of the endometrium into the myometrium, which impacts a large number of childbearing age women. The diagnosis of AM relies on imaging and histological examinations. Although carbohydrate antigen 125 (CA125) has served as a blood indicator for AM diagnosis, its utility is limited to being effective in only approximately half of patients. Currently, there are no reliable blood diagnostic indicators available for AM.

**Methods:**

Data of 23 blood indicators examined for 143 patients with AM and 143 age-matched healthy women were collected, including six sex hormones, two tumor biomarkers, nine routine data, two inflammatory and coagulation indicators, and four lipid-related indicators. Wilcoxon rank-sum test was applied to identify differentially changed indicators (DCIs) for AM *versus* controls. Similarly, Wilcoxon rank-sum test was conducted to determine the DCIs associated with the MRI subtypes of AM. Univariate and multivariate analyses were performed to select the DCIs that might differentiate severe from mild AM. Least absolute shrinkage and selection operator (LASSO) and support vector machine recursive feature elimination (SVMRFE) were used to determine the key DCIs for AM. Logistic regression was carried out to develop a diagnostic model, and the area under the receiver operating characteristic (ROC) curve (AUC) was calculated to evaluate the performance of the model. A nomogram was constructed to predict the risk of AM.

**Results:**

We identified 15 DCIs for AM. Four DCIs were found in all three MRI subtypes. CA125 and estradiol could distinguish severe from mild AM. The hemoglobin (HGB) concentration, lymphocyte percentage, neutrophil count, neutrophil percentage, and testosterone were different between diffuse AM and adenomyoma. Based on these DCIs, neutrophil count, HGB concentration, and high-density lipoprotein (HDL) were selected using the LASSO and SVMRFE methods, which could discriminate AM cases with CA125<35 and ≥35 U/ml from the controls with AUCs of 0.812 and 0.928, respectively. Similarly, CA125, neutrophil count, HGB concentration, and HDL were screened and a diagnostic model built for AM, which could differentiate all AM cases from the controls with an AUC of 0.935 (sensitivity = 0.902, specificity = 0.888).

**Conclusion:**

To our knowledge, these indicators are reported here for the first time as combined biomarkers for the diagnosis of AM. Our findings might provide clues for the pathogenesis research of AM and supply potential blood indicators to assist in its diagnosis.

## 1 Introduction

Adenomyosis (AM) is described as a benign invasion of the endometrium into the myometrium, leading to menorrhagia, anemia, dysmenorrhea, chronic pelvic pain, and infertility (1). The majority of patients with AM are diagnosed at 40–50 years of age. However, younger patients with infertility have been increasingly diagnosed with AM due to improved imaging modalities (2). The prevalence of AM in women at childbearing age ranges from 20% to 34% (3). The actual prevalence may be higher due to its diverse clinical presentations and complexity in accurately diagnosing. Because the symptoms of AM could significantly impact the quality of life of patients, early diagnosis and treatment are essential to alleviate its symptoms and prevent its progression.

AM diagnosis is a significant challenge due to the variability of the symptoms. Histopathology examination is the gold standard for AM diagnosis; however, its application is frequently impractical, particularly in non-surgical patients (4). With advancements in medical imaging, the diagnosis of AM is increasingly based on technologies such as transvaginal sonography (TVS) and magnetic resonance imaging (MRI) (2, 5). TVS serves as the preferred diagnostic method due to its noninvasive nature, accuracy, practicality, and cost-effectiveness (6). However, its ability to detect more extensive or atypical lesions is limited (7). MRI offers superior specificity, particularly in cases with concurrent leiomyoma, making it the preferred method in complex cases (8). However, its high cost and limited availability restrict widespread use (9).

Blood represents a readily accessible source for disease biomarker discovery. Some studies have reported associations between elevated levels of carbohydrate antigen 125 (CA125) and AM (10, 11), but its diagnostic utility remains limited. A level of 35 U/ml as the cutoff for CA125 exhibits controversy and is not endorsed for the screening of asymptomatic women (10). Given that not all patients with AM have a level of CA125 exceeding this threshold (10, 12), it is not sufficient to rely solely on CA125 for AM diagnosis. Despite exploration of S100-A12 and sFRP-4 (13), the red cell distribution width, mean corpuscular hemoglobin concentration, activated partial thromboplastin time, international normalized ratio, antithrombin III (14), and exosome HSP90A, STIP1, TAGLN-2 (15) are associated with AM; however, these markers have yet to achieve validation as reliable diagnostic biomarkers, owing largely to the heterogeneity of AM. Currently, CA125 remains the only blood-based indicator with some diagnostic value, which implies the pressing need to identify more robust and reliable indicators for AM diagnosis.

Therefore, the objective of this study was to identify differentially changed indicators (DCIs) and screen potential diagnostic indicators for AM with/without CA125 using multiple machine learning methods. The findings may provide clues for the pathogenesis research of AM and supply potential blood indicators to assist in its diagnosis.

## 2 Materials and methods

### 2.1 Study design and participants

This study includes patients who initially sought cure for AM and women who underwent physical examination at the Jinhua Maternal and Child Health Care Hospital between August 2020 and December 2024. The medical records of these participants were reviewed, and data on age and blood indicators were collected. Approval was obtained from the Ethics Committee of Jinhua Maternal and Child Health Care Hospital (ethical approval no. 2025QT112).

A total of 784 participants were reviewed, with 286 meeting the inclusion criteria. The specific recruitment details were as follows: 143 patients were diagnosed with AM according to their medical history, clinical symptoms, physical signs, and transvaginal ultrasound examination following the Morphological Uterus Sonographic Assessment (MUSA) criteria (16, 17). Some patients also underwent MRI examination. All of these patients had histopathology examination for AM with a pathologist. Women who had a health checkup at the physical examination department of the hospital were recruited into the control group. Physical examination included blood routine tests, tumor marker tests (CA125 and CA199), ultrasound (transvaginal ultrasound or transabdominal ultrasound), and additional testing including fertility and endocrine testing. Those who might have AM and other gynecological diseases were excluded based on their medical history, clinical symptoms, and ultrasound results. Finally, 143 age-matched healthy women were selected as the control group. The overall inclusion criteria were as follows: 1) aged 25–60 years and age-matched between AM patients and controls; 2) confirmed disease via imaging and histopathology; and 3) access to thorough medical information. The exclusion criteria were: 1) history of hormone therapy within 3 months; 2) with endometriosis and polycystic ovary syndrome; and 3) with gynecological tumors.

For further disease evaluation, 118 patients with AM underwent MRI examination. Patients were divided into four subtypes—MRI-I (*n* = 6), MRI-II (*n* = 19), MRI-III (*n* = 25), and MRI-IV (*n* = 68)—on the basis of MRI geography according to the criteria proposed by Kishi et al. (18). MRI-I AM occurs in the uterine inner layer without affecting the outer structures, MRI-II occurs in the uterine outer layer without affecting the inner structures, MRI-III AM occurs solitarily without any association with structural components, and MRI-IV AM does not meet any of the categorization criteria previously mentioned. Subtypes MRI-I, MRI-II, and MRI-III were thought to be a product of endometrial invasion, endometriotic invasion, and *de novo* metaplasia, respectively. Subtype MRI-IV is a heterogeneous mixture of far-advanced disease. The key parameters of the participants are given in **Table 1**.

**Table 1:** Key parameters of the participants.

Key parameter	AM patients Mean (SD) or *n* (%)	Healthy controls Mean (SD) or *n* (%)
Age (years)	45.8 (6.2)	45.8 (6.2)
BMI (kg/m^2^)	24.1 (3.1)	22.9 (2.7)
MRI subtypes of AM		/
MRI-I	6 (4.2%)	
MRI-II	19 (13.3%)	
MRI-III	25 (17.5%)	
MRI-IV	68 (47.5%)	
None	25 (17.5%)	
Type of AM		/
Diffuse	109 (76.2%)	
Focal	25 (17.5%)	
Diffuse+focal	9 (6.3%)	

AM, adenomyosis; BMI, body mass index.

### 2.2 Study variables

There were 23 blood indicators retrieved from the medical records of the participants for analysis. Among these, luteinizing hormone (LH), follicle-stimulating hormone (FSH), progesterone, estradiol (E2), prolactin (PRL), and testosterone (T) comprised the sex hormone-related indicators. CA125 and CA199 were the tumor biomarkers. Red blood cell (RBC) count, hemoglobin (HGB) concentration, white blood cell (WBC) count, neutrophil count, neutrophil percentage, lymphocyte count, lymphocyte percentage, monocyte count, and monocyte percentage were the blood routine indices. High-sensitivity C-reactive protein (hs-CRP) and D-dimer were the inflammatory and coagulation indicators. Total cholesterol (TC), triglycerides (TG), high-density lipoprotein (HDL), and low-density lipoprotein (LDL) were the blood lipid-related indicators.

### 2.3 Statistical analyses

Statistical analysis was performed using R software (version 4.4.0). The Shapiro–Wilk test was used to examine whether the levels of each indicator followed a normal distribution. Due to these data not complying with a normal distribution, they are presented as median (25th–75th quantile). The Wilcoxon rank-sum test combined with the false discovery rate (FDR) was applied to identify the DCIs for disease. Fold change (FC) was further calculated for each indicator to determine whether it is upregulated or downregulated in disease *versus* the control. For the MRI subtypes of AM, the Wilcoxon rank-sum test was also used to determine the DCIs for each subtype relative to the control and the DCIs for severe AM relative to mild AM. Univariate and multivariate regressions were performed to select the DCIs that might differentiate severe from mild AM. In addition, the Wilcoxon rank-sum test was used to determine DCIs in diffuse AM *versus* adenomyoma. Least absolute shrinkage and selection operator (LASSO) and support vector machine recursive feature elimination (SVMRFE) were performed to select the key indicators for AM. LASSO analysis was conducted using the “glmnet” package in R, with the specific parameters as follows: family = “binomial,” *n*-fold = 10, and *λ* = “lambda.1se.” SVMRFE was carried out using the “e1071” package of R with the *n*-fold set to 10. Logistic regression algorithm was carried out to establish a diagnostic model for AM based on selected key indicators. The area under the receiver operating characteristic (ROC) curve (AUC) was calculated to evaluate the accuracy of the model. A nomogram was developed to predict the risk of AM using the “rms“ package in R. Calibration curves were generated to estimate the prediction efficacy of the nomogram. Decision curve analysis (DCA) was conducted to exhibit the clinical utility of the nomogram. Statistical significance was set to adjust-*p* < 0.05 as the cutoff.

## 3 Results

### 3.1 General characteristics of the participants

Overall, 286 participants were included in this retrospective study, comprising 143 patients with AM and 143 age-matched healthy controls. The median (25th–75th quantile) age for each group of participants was 46 (42–51) years. There were 23 blood indicators examined across all participants. The median (25th–75th quantile) levels of these indicators are shown in **Table 2**. Differential analysis identified 15 DCIs for AM *versus* controls, including six upregulated and nine downregulated indicators.

**Table 2:** Levels of the blood indicators of participants.

Blood indicator	AM patients	Healthy controls	*p*-adjusted
LH (IU/L)	5.54 (3.65–11.69)	6.40 (3.71–18.03)	0.156
FSH (IU/L)	7.72 (4.49–13.09)	8.98 (5.50–16.05)	0.068
Progesterone (nmol/L)	0.52 (0.33–1.23)	0.69 (0.42–4.12)	0.004 *
Estradiol (pmol/L)	88.02 (49.86–157.46)	90.33 (47.92–159.47)	0.728
PRL (ng/mL)	11.96 (8.66–16.55)	10.35 (7.72–15.41)	0.137
Testosterone (nmol/L)	18.93 (14.61–25.35)	21.77 (18.45–28.19)	<0.001*
CA125 (U/ml)	43.74 (24.77–93.68)	11.17 (7.64–16.22)	<0.001*
CA199 (U/ml)	17.93 (8.29–32.61)	10.70 (4.27–17.91)	<0.001*
WBC count (10^9^/L)	7.05 (5.07–9.74)	5.24 (4.32–6.27)	<0.001*
Neutrophil count (10^9^/L)	4.99 (3.14–7.43)	3.20 (2.50–3.84)	<0.001*
Neutrophil percentage (%)	72.40 (62.25–80.85)	61.00 (56.35–67.00)	<0.001*
Lymphocyte count (10^9^/L)	1.33 (1.04–1.70)	1.59 (1.30–1.90)	<0.001*
Lymphocyte percentage (%)	20.80 (13.45–29.40)	30.60 (25.30–36.25)	<0.001*
RBC count (10^12^/L)	3.98 (3.65–4.27)	4.28 (4.03–4.49)	<0.001*
HGB concentration (g/L)	105 (87.00–120.50)	127 (117.50–136.00)	<0.001*
Monocyte count (10^9^/L)	0.36 (0.28–0.53)	0.29 (0.22–0.34)	<0.001*
Monocyte percentage (%)	5.40 (4.55–6.55)	5.50 (4.40–6.35)	0.728
hs-CRP (mg/L)	0.52 (0.27–1.25)	0.66 (0.35–1.11)	0.488
D-dimer (mg/L FEU)	330 (270–390)	320 (260–375)	0.204
TC (mmol/L)	3.93 (3.40–4.44)	4.24 (3.78–4.82)	<0.001*
TG (mmol/L)	1.06 (0.78–1.48)	1.05 (0.79–1.58)	0.588
HDL (mmol/L)	1.15 (0.99–1.36)	1.41 (1.23–1.68)	<0.001*
LDL (mmol/L)	2.59 (2.05–3.20)	2.79 (2.34–3.33)	0.009*

AM, adenomyosis; LH, luteinizing hormone; FSH, follicle-stimulating hormone; PRL, prolactin; CA125, carbohydrate antigen 125; WBC, white blood cells; RBC, red blood cells; HGB, hemoglobin; hs-CRP, high-sensitivity C-reactive protein; TC, total cholesterol; TG, triglycerides; HDL, high-density lipoprotein; LDL, low-density lipoprotein.

* indicates that the indicator is significantly changed in AM cases versus controls.

### 3.2 Identification of DCIs for the MRI subtypes of AM

Among the 143 patients with AM, 118 underwent MRI examination for disease evaluation. According to the findings of MRI geography, patients with AM were classified into four subtypes: MRI-I (*n* = 6), MRI-II (*n* = 19), MRI-III (*n* = 25), and MRI-IV (*n* = 68). In order to determine the DCIs associated with the MRI subtypes of the AM patients, differential analysis was carried out for each MRI subtype relative to the controls. Subtype MRI-IV was not included in the analysis because this subtype consisted of heterogeneous mixtures of advanced cases of subtypes MRI-I, MRI-II, and MRI-III. Thereafter, 10, 11, and 13 DCIs were identified for subtypes MRI-I, MRI-II, and MRI-III, respectively. Among these DCIs, CA125, neutrophil percentage, lymphocyte percentage, and HGB concentration were found in all three subtypes. Lymphocyte count, hs-CRP, and D-dimer were specific in subtype MRI-I, while LH, FSH, and testosterone were specific in subtype MRI-III. There were no DCIs specific in subtype MRI-II. The average levels of these DCIs for the MRI subtypes and the controls are shown in **Table 3**.

**Table 3:** Levels of the differentially changed indicators (DCIs) for the MRI subtypes of adenomyosis (AM) and controls.

DCI	Control	MRI-I		MRI-II		MRI-III	
	Expr	Expr	*p*-adj.	Expr	*p*-adj.	Expr	*p*-adj.
CA125	11.50	36.64	0.016*	39.20	5.44E−7*	59.83	2.11E−12*
Neutrophil percentage	61.82	74.02	0.012*	71.05	1.72E−3*	73.51	3.19E−7*
Lymphocyte percentage	30.52	20.08	0.016*	21.53	1.09E−3*	20.05	8.58E−7*
HGB concentration	126.62	105.00	0.040*	91.53	5.81E−8*	111.96	1.04E−3*
Lymphocyte count	1.59	1.17	0.040*	1.38	0.102	1.44	0.183
hs-CRP	0.73	0.22	0.006*	0.50	0.107	0.97	0.412
D-dimer	349.00	292.00	0.040*	332.00	0.306	346.25	0.404
LH	10.66	6.49	0.757	9.29	0.742	4.63	0.007*
FSH	8.85	10.19	0.897	7.91	0.338	6.95	0.015*
Testosterone	23.30	21.01	0.466	20.82	0.383	18.27	2.85E−4*

*CA125*, carbohydrate antigen 125; *HGB*, hemoglobin; *hs-CRP*, high-sensitivity C-reactive protein; *LH*, luteinizing hormone; *FSH*, follicle-stimulating hormone.

* indicates that the indicator is significantly changed in AM cases versus controls.

**Figure 1:**
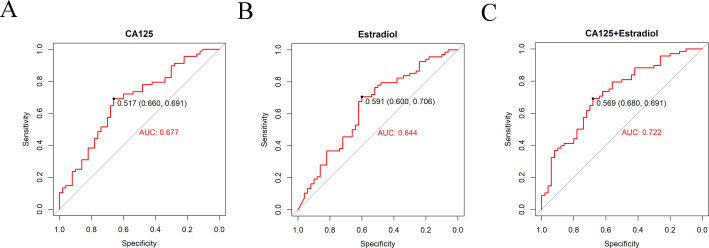
Receiver operating characteristic (ROC) curve analysis on differentially changed indicators (DCIs) to evaluate the ability in distinguishing severe from mild adenomyosis (AM). **(A–C)** ROC curve analysis on carbohydrate antigen 125 (CA125) **(A)**, estradiol **(B)**, and CA125 and estradiol **(C)**.

Furthermore, patients with AM were classified into two groups based on the MRI subtype: patients with MRI-I, MRI-II, and MRI-III as the mild group and patients with MRI-IV as the severe group. Differential analysis revealed that the level of CA125 was significantly upregulated (FC = 1.43, *p* = 0.001) while estradiol was downregulated (FC = 0.70, *p* = 0.008) in the severe group *versus* the mild group. In addition, univariate and multivariate analyses were performed to assess whether the two indicators could distinguish patients with mild AM from those with the severe, as shown in **Table 4**. The univariate analyses showed that CA125 and estradiol could differentiate severe from mild AM, with AUC values of 0.677 (sensitivity = 0.691, specificity = 0.660) and 0.644 (sensitivity = 0.706, specificity = 0.600), respectively (**Figures 1A, B**). The multivariate analysis showed that CA125 and estradiol could significantly discriminate severe from mild AM. The ROC curve analysis showed an AUC of 0.722 (sensitivity = 0.691, specificity = 0.680) (**Figure 1C**).

**Table 4:** Univariate and multivariate analyses of severe and mild adenomyosis (AM).

Variable	Univariate		Multivariate	
	OR (95%CI)	*p*	OR (95%CI)	*p*
CA125	1.013 (1.004–1.022)	0.0053	1.014 (1.005–1.024)	0.0036
Estradiol	0.992 (0.986–0.997)	0.0058	0.991 (0.984–0.997)	0.0037

*CA125*, carbohydrate antigen 125.

### 3.3 Profiles of DCIs in patients with diffuse AM and adenomyoma

**Figure 2:**
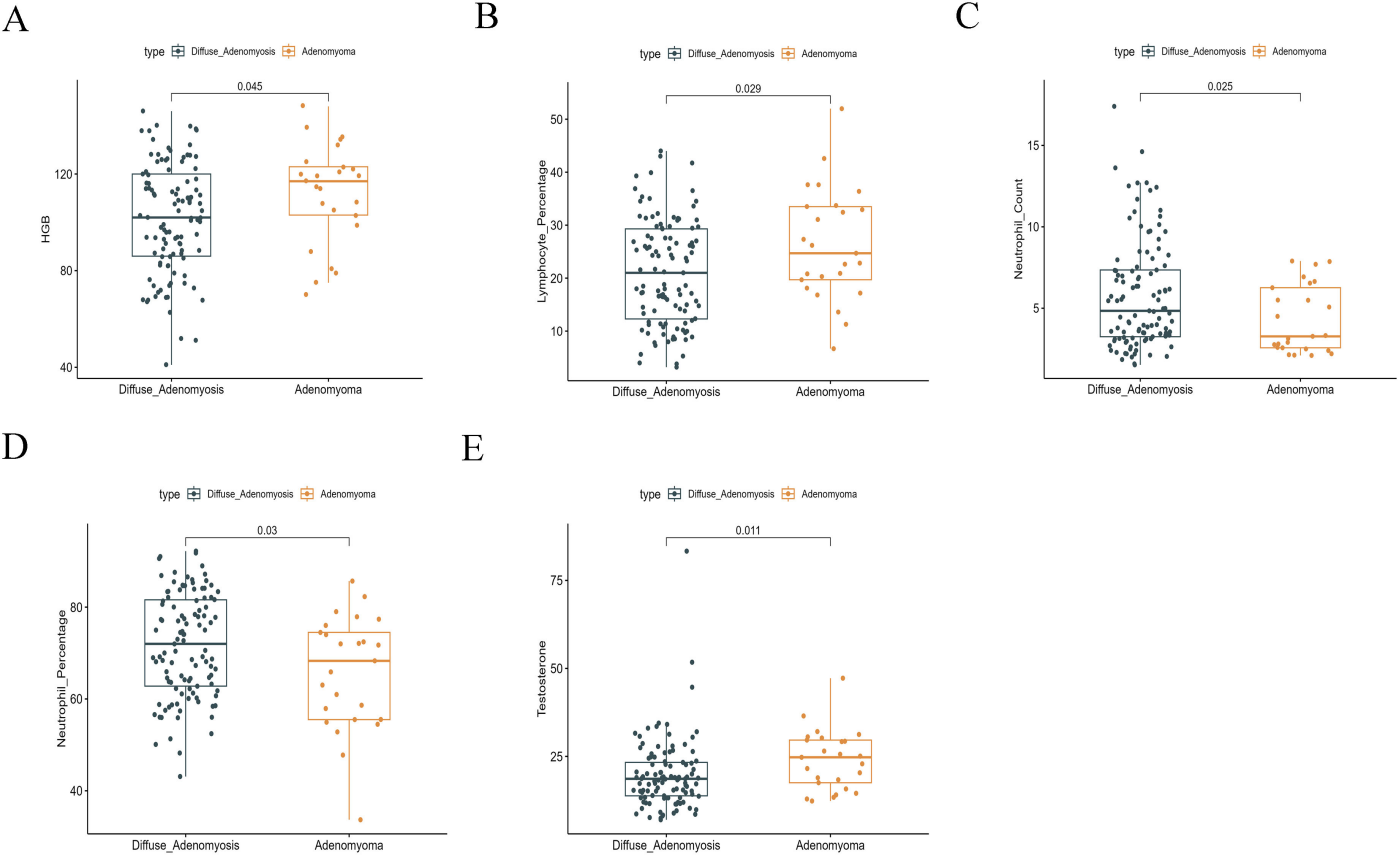
The levels of the differentially changed indicators (DCIs) in adenomyoma (AM) *versus* diffuse AM. **(A)** Hemoglobin (HGB). **(B)** Lymphocyte percentage. **(C)** Neutrophil count. **(D)** Neutrophil percentage. **(E)** Testosterone.

In general, AM is classified into two primary subtypes, namely diffuse AM and focal AM (known as adenomyoma). Based on the pathological findings, there were 109 patients with diffuse AM, 25 with adenomyoma, and nine with a combination of these two types. To reveal the profiles of the DCIs in patients with diffuse AM and adenomyoma, Wilcoxon rank-sum test was conducted to evaluate the levels of the DCIs in these two subtypes. It was found that three DCIs (HGB concentration, lymphocyte percentage, and testosterone) were upregulated and two (neutrophil count and neutrophil percentage) were downregulated in adenomyoma in contrast to diffuse AM, as illustrated in **Figure 2**.

### 3.4 Diagnostic blood indicators other than CA125 for AM patients

**Figure 3:**
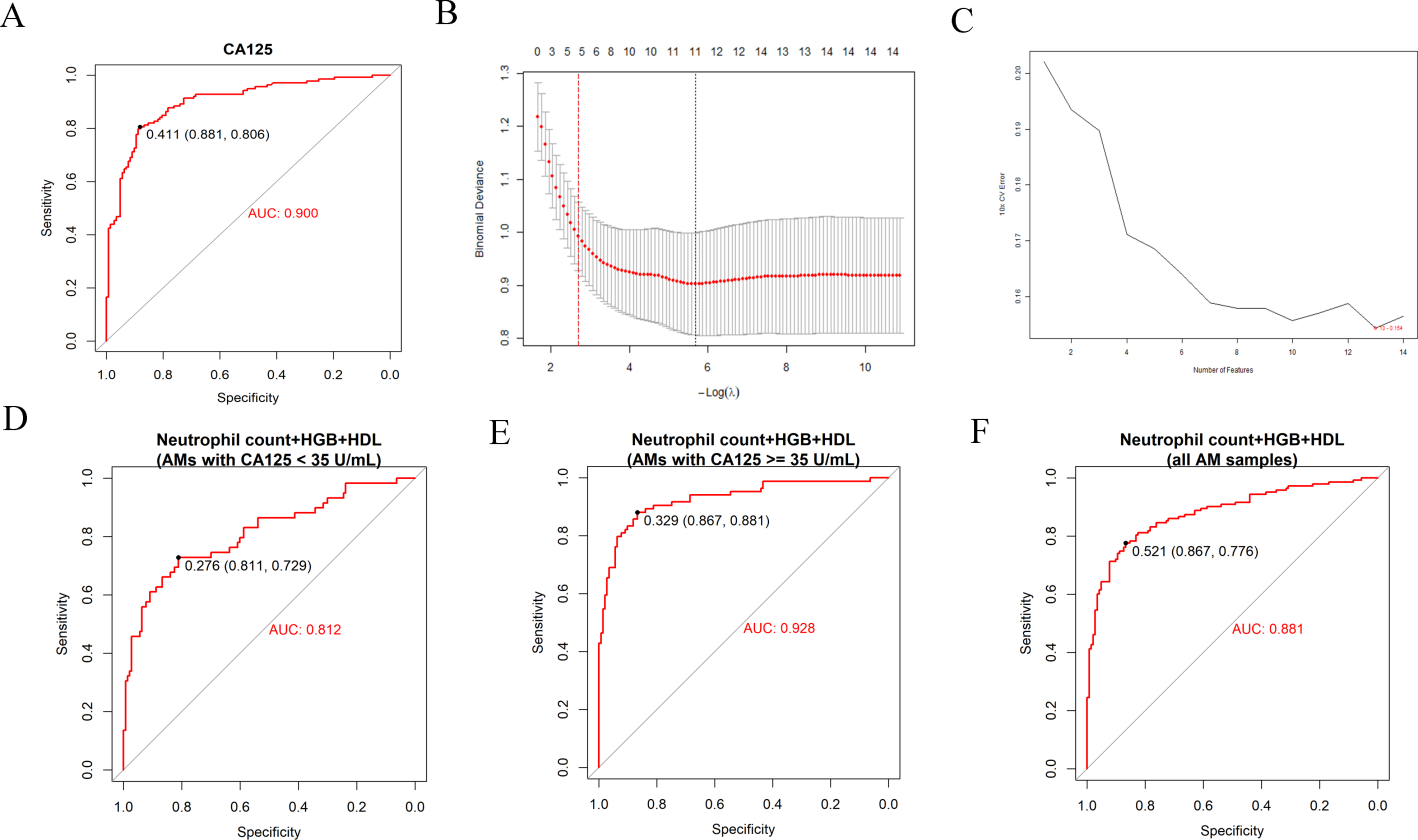
Least absolute shrinkage and selection operator (LASSO) and support vector machine recursive feature elimination (SVMRFE) were used to screen the key differentially changed indicators (DCIs) of adenomyosis (AM), and receiver operating characteristic (ROC) was used to evaluate the efficacy of the diagnostic indicators in distinguishing AM from controls. **(A)** ROC curve analysis of carbohydrate antigen 125 (CA125). **(B)** Number of selected indicators at different lambda for LASSO. **(C)** Number of selected indicators at different errors for SVMRFE. **(D, E)** ROC curve analysis of three indicators for AM cases with CA125 < 35 U/ml **(D)** and with CA125 ≥ 35 U/ml **(E)** *vs*. controls. **(F)** ROC curve analysis of three indicators for all AM cases *vs*. controls.

CA125 has been used as an auxiliary diagnostic biomarker for AM, with 35 U/ml as the established cutoff level. In our data, CA125 could exactly discriminate AM patients from healthy controls, with an AUC of 0.900; however, the optimal threshold was at 21.905 U/ml with a sensitivity of 0.806 and a specificity of 0.881 (**Figure 3A**). Further analysis revealed that 59 patients with AM had levels of CA125 less than 35 U/ml, accounting for 41.26% (59/143) of the total AM patients. Therefore, it is essential to determine other diagnostic blood indicators for patients with AM (CA125 < 35 U/ml).

In order to discover diagnostic indicators suitable for AM patients with CA125 < 35 U/ml, two different machine learning algorithms were used to screen the key blood indicators. Five key DCIs were determined using the LASSO method: neutrophil count, lymphocyte percentage, RBC count, HGB concentration, and HDL (**Figure 3B**). Moreover, 13 key DCIs were obtained through the SMRFE method, with minimal error of 0.154 (**Figure 3C**). The intersection of these two groups of key indicators comprises the neutrophil count, lymphocyte percentage, RBC count, HGB concentration, and HDL. Furthermore, the multivariate regression analysis showed that HDL (OR = 0.160, 95%CI = 0.038–0.583, *p* = 0.008) and HGB concentration (OR = 0.973, 95%CI = 0.948–0.998, *p* = 0.034) were significantly associated with AM. Neutrophil count (OR = 1.298, 95%CI = 0.999–1.740, *p* = 0.063) was also related to AM, but with a relatively not strong statistical significance. Based on these three key DCIs, a diagnostic model was constructed using logistic regression. The ROC curve analysis showed that the AUC was 0.812 (sensitivity = 0.729, specificity = 0.811), indicating that the diagnostic model had good performance in distinguishing AM cases (CA125 < 35 U/ml) from the controls (**Figure 3D**). Furthermore, HDL, HGB concentration, and neutrophil count were also able to discriminate AM cases (CA125 > 35 U/ml) from the controls, with an AUC of 0.928 (sensitivity = 0.881, specificity = 0.867) (**Figure 3E**). For all AM patients, the three key DCIs could discriminate AM from controls with an AUC of 0.881 (sensitivity = 0.867, specificity = 0.776) (**Figure 3F**).

### 3.5 Diagnostic blood indicators including CA125 for AM patients

**Figure 4:**
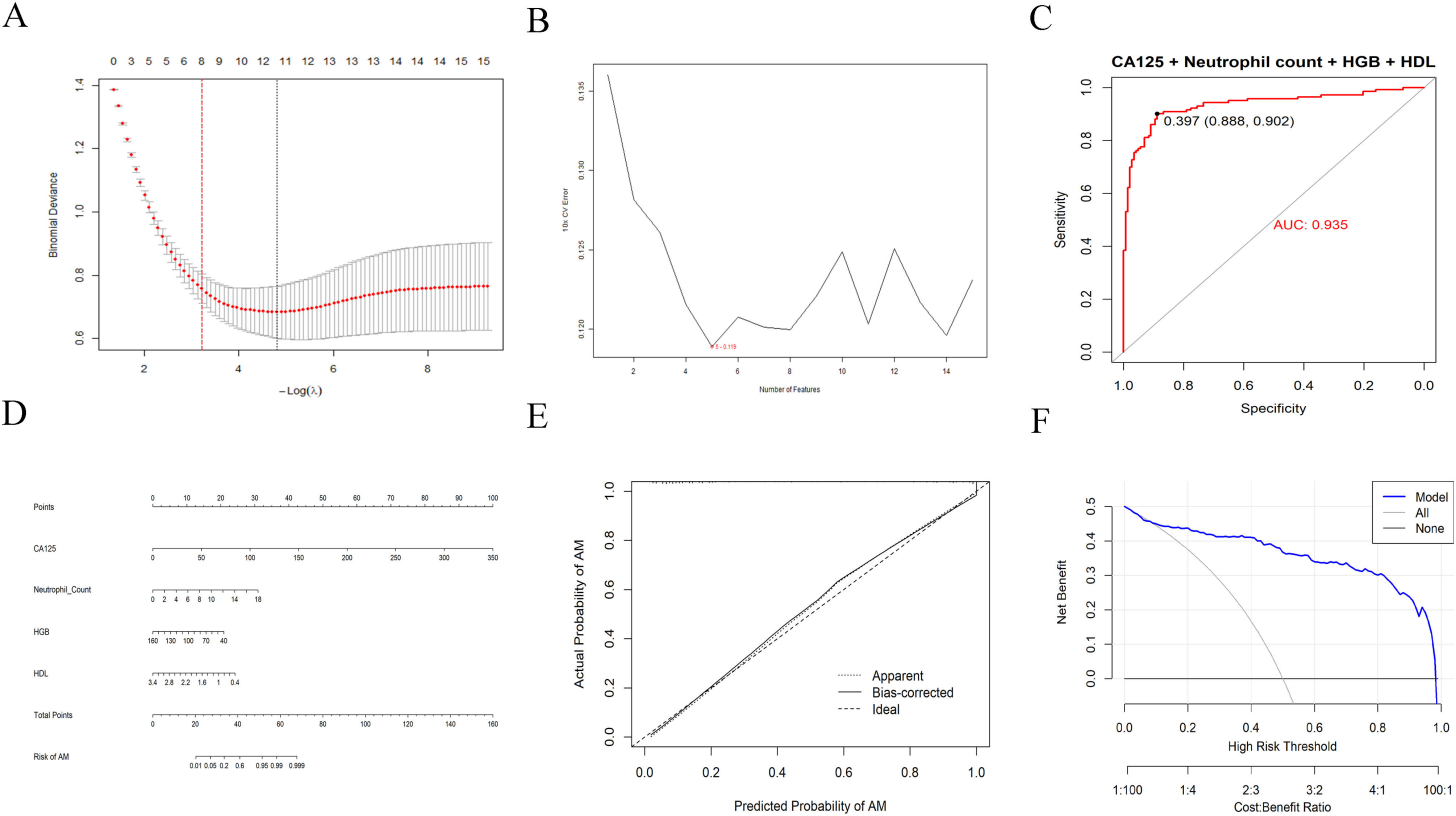
Least absolute shrinkage and selection operator (LASSO) and support vector machine recursive feature elimination (SVMRFE) were used to screen the key differentially changed indicators (DCIs) for adenomyosis (AM), and receiver operating characteristic (ROC) curve analysis was used to evaluate the performance of the diagnostic model. **(A)** Number of selected indicators at different lambda for LASSO. **(B)** Number of selected indicators at different errors for SVMRFE. **(C)** ROC curve analysis of four indicators including carbohydrate antigen 125 (CA125). **(D)** A nomogram model constructed to predict the risk of AM. **(E)** Calibration curve of the nomogram given the performance of the model. **(F)** Decision curve analysis (DCA) exhibiting the clinical utility of the nomogram.

Due to the limitations of CA125 in the auxiliary diagnosis of AM mentioned above, it is essential to determine a combination of DCIs including CA125 to improve the diagnostic accuracy in AM. Using LASSO analysis, eight DCIs were identified for AM cases compared with the controls: CA125, CA199, neutrophil count, monocyte count, progesterone, lymphocyte percentage, HGB concentration, and HDL (**Figure 4A**). Using SVMRFE analysis, five other DICs were determined for AM *versus* controls: CA125, HGB concentration, HDL, lymphocyte count, and neutrophil count (**Figure 4B**). Comparative analysis showed that CA125, neutrophil count, HGB concentration, and HDL were the consistent indicators obtained by the two machine learning methods. Furthermore, a diagnostic model was established using logistic regression based on these four indicators. The ROC curve analysis showed that it could distinguish AM patients from healthy controls with an AUC of 0.935 (sensitivity = 0.888, specificity = 0.902), indicating better performance than that of CA125 (**Figure 4C**). The nomogram was constructed to predict the risk of AM by combining the four indicators (**Figure 4D**). The contribution of each indicator to the model is represented by the length of the line in the nomogram. The calibration curve showed agreement between the actual and the predicted probabilities, demonstrating the good predictive performance of the nomogram (**Figure 4E**). DCA exhibited that the nomogram offered greater benefits relative to either “all” or “none” across a threshold probability ranging from 0.1 to 0.98 (**Figure 4F**).

## 4 Discussion

There were 286 participants in this study, including 143 patients with AM and 143 healthy women. The age of each group was matched and no difference exists. The median (25th–75th quantile) age for each group was 46 (42–51) years. There are several clinical benefits of diagnosing AM in this population. Firstly, AM has been thought to occur most likely during ages 40–50 years, and early diagnosis and treatment of the disease could alleviate symptoms and enhance the quality of life. Secondly, the diagnostic indicators identified from this population may be applied to the diagnosis of childbearing patients, which would be beneficial for the early diagnosis and treatment of young patients, thereby improving their fertility rates.

Thereafter, the data of 23 blood indicators from all participants were collected and analyzed. There were 15 DCIs identified for AM, of which CA125, CA199, WBC, neutrophil count, neutrophil percentage, and monocyte count were upregulated, while progesterone, testosterone, lymphocyte count, lymphocyte percentage, RBC count, HGB concentration, TC, HDL, and LDL were downregulated. Among them, CA125 and CA199 have been reported to be upregulated in AM in previous studies (10, 19–21). In particular, CA125 has served as a diagnostic biomarker for AM and is positively correlated with its severity (12, 21, 22). In addition, a decreased progesterone may induce abnormal pathophysiological changes in the endometrium, which could lead to the development of gynecological disorders such as AM (23). AM has been reported to exhibit a uniquely estrogen-driven inflammatory process and progesterone resistance (1). Lymphocytes are tightly associated with inflammation. Numerous observations have highlighted the association between inflammation and immune response, as well as AM development (24). The platelet-to-lymphocyte ratio is a commonly available biomarker of inflammation (25). RBC count has been reported to have a moderate negative correlation with the level of CA125 in patients with endometriosis (26), suggesting that it may be associated with AM. HGB concentration has been proven to be one of the key distinguishing indices for the differentiation of AM and endometriosis (14). The levels of HGB have been used primarily to assess menorrhagia (27). The level of HDL-C was found to be more likely lower in AM (28), inferring that HDL is associated with AM.

Furthermore, AM could be classified into four subtypes based on MRI examination. Differential analysis revealed that the CA125 level, neutrophil percentage, lymphocyte percentage, and HGB concentration were significantly changed in all subtypes, i.e., MRI-I, MRI-II, and MRI-III, inferring that inflammation and immune response may play an important role in all subtypes of AM. Several indictors were found to be differentially changed only in MRI-I or MRI-III, implying that they may be associated with the development of these subtypes. More samples are needed for research and verification of the findings. Moreover, CA125 and estradiol could discriminate severe from mild AM with an AUC of 0.722. CA125 has been reported to have an association with AM progression in previous studies (12, 21, 22, 29). Estradiol was found significantly downregulated in the severe group *versus* the mild group, which is consistent with the report that aberrant gene expression of AM is related to pathways that favor a decreased estradiol metabolism (1). Estradiol modulates gap junctions during AM, implying that it plays an important role in the etiology of AM (30).

Clinically, AM is typically categorized into diffuse and focal subtypes (31). To further elucidate the molecular underpinnings of these distinct subtypes, the levels of the previously identified DCIs were analyzed. Compared to patients with diffuse AM, those with adenomyoma exhibit significantly elevated levels of HGB, lymphocyte percentage, and testosterone in the blood, along with abnormally decreased neutrophil count and neutrophil percentage. This suggests that adenomyoma patients may have different immune response profiles from diffuse AM patients. In addition, HGB and testosterone may be involved in the formation process of adenomyoma, the potential mechanism of which has not been studied. Some studies discovered that testosterone could affect the glucose metabolism in the endometrium (32).

Considering that it is controversial to apply CA125 using 35 U/ml as the threshold for AM diagnosis, it is essential to determine other blood indicators for AM. Using the LASSO and SVMRFE methods, HDL, HGB concentration, and neutrophil count were identified to have the ability to distinguish AM cases (CA125 < 35 U/ml) from controls with an AUC of 0.812, to discriminate AM (CA125 ≥ 35 U/ml) from controls with an AUC of 0.928, and to differentiate all AM cases from controls with an AUC of 0.881, implying the possibility to diagnose AM by blood routine examination as an auxiliary. Using similar methods, CA125, HDL, HGB concentration, and neutrophil count were determined to display good performance in discriminating AM cases from controls with an AUC of 0.935, suggesting their potential to become diagnostic indicators for AM. To our knowledge, these indicators are reported here for the first times as combined biomarkers for AM diagnosis. Furthermore, these findings need large-scale validation in further research.

This study is subject to several limitations. Firstly, avoiding selection bias was a challenge. Although AM patients and controls were age-matched, there was still some bias on the sample selection across groups. Secondly, this is a single-center study, which lacked external data for verification. Thirdly, the constrained sample size may potentially compromise the robustness of our findings. Consequently, it is essential to have a larger patient cohort to confirm the efficiency and specificity of the findings.

In conclusion, this study identified 15 DCIs for AM *versus* controls. CA125 and estradiol could differentiate severe AM from mild AM. The HGB level, neutrophil percentage, lymphocyte percentage, neutrophil count, and testosterone level were different between diffuse and focal AM. Furthermore, neutrophil count, HGB concentration, and HDL could discriminate AM cases with CA125<35 and ≥35U/ml from the controls with AUCs of 0.812 and 0.928, respectively. Similarly, four DCIs (CA125, HDL, HGB concentration, and neutrophil count) were predicted as potential diagnostic blood indicators for AM *versus* controls, with good performance (AUC = 0.935). These findings may provide clues for the pathogenesis research of AM and supply potential blood indicators for the auxiliary diagnosis of AM.

## Data Availability

The raw data supporting the conclusions of this article will be made available by the authors, without undue reservation.
